# Fractalkine/CX3CL1 engages different neuroprotective responses upon selective glutamate receptor overactivation

**DOI:** 10.3389/fncel.2014.00472

**Published:** 2015-01-21

**Authors:** Clotilde Lauro, Myriam Catalano, Eleonora Di Paolo, Giuseppina Chece, Ida de Costanzo, Flavia Trettel, Cristina Limatola

**Affiliations:** ^1^Department of Physiology and Pharmacology, Istituto Pasteur Fondazione Cenci Bolognetti, Sapienza University of RomeRome, Italy; ^2^Istituto di Ricovero e Cura a Carattere Scientifico NeuroMedPozzilli, Italy; ^3^Department of Biosciences, University of MilanMilan, Italy

**Keywords:** CX3CL1, NMDA, A_2A_R, D-serine, neuroprotection, excitotoxicity

## Abstract

Neuronal death induced by overactivation of N-methyl-d-aspartate receptors (NMDARs) is implicated in the pathophysiology of many neurodegenerative diseases such as stroke, epilepsy and traumatic brain injury. This toxic effect is mainly mediated by NR2B-containing extrasynaptic NMDARs, while NR2A-containing synaptic NMDARs contribute to cell survival, suggesting the possibility of therapeutic approaches targeting specific receptor subunits. We report that fractalkine/CX3CL1 protects hippocampal neurons from NMDA-induced cell death with a mechanism requiring the adenosine receptors type 2_A_ (A_2A_R). This is different from CX3CL1-induced protection from glutamate (Glu)-induced cell death, that fully depends on A_1_R and requires in part A_3_R. We show that CX3CL1 neuroprotection against NMDA excitotoxicity involves D-serine, a co-agonist of NR2A/NMDAR, resulting in cyclic AMP-dependent transcription factor cyclic-AMP response element-binding protein (CREB) phosphorylation.

## Introduction

Neuroprotection is a complex procedure aimed at preservation of neuronal structure and function upon toxic insults. Glutamate (Glu)-induced excitotoxicity is one of the most important mechanism known to trigger cell death in CNS disorders: over-excitation of Glu receptors, principally N-methyl-d-aspartate receptors (NMDARs), allows for an uncontrolled increase of intracellular Ca^2+^ and the dysregulation of intracellular pathways leading to cell death (Sattler and Tymianski, 2000, 2001). Nevertheless, depending on the receptor subtype involved, NMDA stimulation may exert protective effects. Low levels of Glu activate NMDARs in preconditioning experiments (Liu et al., 2009), resulting protective against a subsequent stronger insult. This is achieved through multifactorial mechanisms involving the rapid adaptation of the voltage-dependent Ca^2+^ channels (Shimazaki et al., 1998), the release of brain derived neurotropic factor (BDNF; Blondeau et al., 2001), the activation of phosphatidylinositol 3-kinase (PI3K)/Akt/glycogen synthase kinase 3-beta signaling (Hetman and Xia, 2000; Papadia et al., 2005; Soriano et al., 2006), extracellular signal-regulated kinase (ERK 1/2), the inactivation of c-Jun N-terminal kinase (JNK) and cyclic-AMP response element-binding protein (CREB; Navon et al., 2012). The NMDAR is a pentameric protein with different subunit composition that changes during development (Monyer et al., 1994). In adulthood, NMDARs with specific subunit composition have different cellular and subcellular distribution in the CNS (Ikeda et al., 1992; Kutsuwada et al., 1992; Monyer et al., 1992). In particular, NR2A and NR2B subunits are abundantly expressed in the hippocampusd (Monyer et al., 1994; Laurie et al., 1997) with a preferential extrasynaptic localization of NR1/NR2B and a synaptic localization of NR1/NR2A/NR2B in the forebrain (Stocca and Vicini, 1998; Tovar and Westbrook, 1999; Thomas et al., 2006). Several studies demonstrated that NMDAR localization correlates with opposite functions: extrasynaptic NMDARs are linked to damage-induced toxicity while synaptic NMDARs provide neuroprotection (Chen et al., 2008; Hardingham and Bading, 2010), activating neuroprotective pathways such as Akt, ERK1/2 (Hardingham et al., 2001a; Papadia et al., 2005; Wang et al., 2013), and CREB (Hardingham et al., 2001b, 2002). NMDAR functions are known to be modulated by co-factors such as glycine and D-serine (Johnson and Ascher, 1987; Matsui et al., 1995). While glycine preferentially acts on extrasynaptic NMDARs, D-serine binds with higher affinity to synaptic NMDARs (Madry et al., 2007; Papouin et al., 2012). We have previously shown that fractalkine/CX3CL1 modulates NMDA-mediated synaptic transmission in the hippocampal CA1 region through the activity of the adenosine receptors type 2_A_ (A_2A_R) and the release of D-serine from glia (Scianni et al., 2013). Since it is known that CX3CL1 is able to counteract excitotoxic damage (Deiva et al., 2004; Limatola et al., 2005) in the present paper we investigated the effects of CX3CL1 on NMDA-mediated neurotoxicity and demonstrated that CX3CL1 is neuroprotective with mechanisms requiring the presence of A_2A_R and extracellular D-serine, leading to CREB phosphorylation.

## Materials and methods

### Materials

Recombinant human CX3CL1 was from Calbiochem/Merck (Nottingham, UK); D-serine was from Ascent Scientific (Bristol, UK); rabbit anti-phospho TrkB (Tyr 515) was from Abcam (Cambridge, UK), rabbit anti-phospo CREB (Ser 133) and rabbit anti-PARP were from Cell Signaling (Danvers, Ma, USA); secondary antibodies were from DAKO (Milan, Italy); culture media were from Invitrogen Life Technologies (San Giuliano Milanese, Italy); 3-[4,5-Dimethylthiazol-2-yl]-2,5-diphenyltetrazolium bromide (MTT), *in vitro* Toxicology Assay Kit Lactic Dehydrogenase based (LDH assay), catalase, D-amino acid oxidase (DAAO), poly-L-lysine, rabbit anti-actin antibody and all the other reagents were from Sigma-Aldrich (Milan, Italy).

### Animals and cell lines

Procedures using laboratory animals were in accordance with the international guidelines on the ethical use of animals from the European Communities Council Directive of 24 November 1986 (86/609/EEC). A_1_R^−/−^ (Johansson et al., 2001) A_2A_R^−/−^ (Chen et al., 1999) and A_3_R^−/−^ (Salvatore et al., 2000) mice were backcrossed at least 10 times on a C57BL/6 background.

### Hippocampal neuronal cultures

Primary hippocampal neuronal cultures were prepared from 0–2-day-old (p0–p2) C57BL/6 (WT), A_1_R^−/−^, A_2A_R^−/−^ and A_3_R^−/−^ mice. Briefly, after careful dissection from diencephalic structures, the meninges were removed and hippocampal tissues chopped and digested for 20 min at 37°C in 0.025% trypsin and Hank’s balanced salt solution (HBSS). Cells were washed twice with HBSS to remove the excess of trypsin, mechanically dissociated in minimal essential medium (MEM) with Earl’s Salts and GLUTAMAX supplemented with 10% dialyzed and heat inactivated fetal bovine serum (FBS), 100 μg/ml gentamycin, and 25 mM KCl. Cells were plated at a density of 2 × 10^5^ in the same medium on poly-L-lysine (100 mg/ml)-coated plastic 24-well dishes. After 1–2 h, the medium was replaced with serum-free Neurobasal/B27. Cells were kept at 37°C in 5% CO_2_ for 11–12 days with twice a week medium replacement (1:1 ratio). At this time point we have 2 × 10^5^ ± 0.05 × 10^5^ alive cells (which corresponds to about 85% of initially plated cells); no significant differences were obtained in the number of living cells in hippocampal preparations obtained from the brains of WT and genetically modified mice. With this method we obtained 60–70% neurons, 30–35% astrocytes, 4–8% microglia, as determined with β-tubulin III, GFAP, and IBA-I staining (Lauro et al., 2010).

### Excitotoxicity experiments

Before stimulation, medium conditioned by cultured cells was removed and stored for later use. To induce excitotoxicity, hippocampal neuronal cultures were stimulated in Locke’s buffer (in mM: 154 NaCl, 5.6 KCl, 3.6 NaHCO3, 5 HEPES, 2.3 CaCl2, 5,6 glucose, 10 glycine pH 7.4) with 100 μM Glu or 200 μM NMDA alone or together with 100 nM CX3CL1 or D-serine at different concentrations (from 10 nM to 10 μM) or vehicle, for 30 min. For excitotoxicity experiments with DAAO hippocampal cultures were pretreated with DAAO (0.1 U/ml) and catalase (300 U/ml) for 1 h and then stimulated with Glu (100 μM) or NMDA (200 μM) in the presence or in the absence of CX3CL1 100 nM or D-serine 100 nM. Following stimulation, cells were washed in Locke’s buffer and re-incubated in the previously stored conditioned medium for additional 16 h. Under these experimental conditions Glu and NMDA induced about 40–50% of cell death in comparison with untreated control cultures. This corresponds to about 70% of neuronal death (Lauro et al., 2010) for necrotic and apoptotic processes (Bonfoco et al., 1995; Nicotera et al., 1999). To evaluate neuron viability cells were treated with detergent-containing buffer (0.05% ethyl hexadecyl dimethylammonium bromide, 0.028% acetic acid, 0.05% Triton X-100, 0.3 mM NaCl, 0.2 mM MgCl2, in PBS pH 7.4) and the remaining nuclei were counted in a hemacytometer, as already described (Volontè et al., 1994). In all figures data were expressed as percentage of viable cells taking as 100% those present in control conditions. To evaluate viability, cells were also analyzed by the MTT assay: in detail, 5 mg/mL MTT was added 1:10 to the cell medium and incubated for 2 h at 37°C; the medium was aspired, cells were treated with DMSO and incubated at 37°C for 10 min. Samples were then analyzed with a microplate reader at 490 nm and 630 nm to subtract background. Data were expressed as % of cell viability, taking as 100% viability in untreated cells.

### LDH activity

To measure *in vitro* citotoxicity due to NMDA, released LDH was measured by LDH assay kit (SIGMA) according to manifacture procedure. Data were expressed as % of extracellular LDH activity in treated cells (NMDA or NMDA/CX3CL1) vs. untreated cells.

### Western blot analysis

Hippocampal neuronal cultures were plated at a density of 2 × 10^5^ on poly-L-lysine (100 mg/ml)-coated plastic 24-well dishes, incubated for 2 h in Locke’s buffer and stimulated for 15 min with CX3CL1 (100 nM), D-serine (100 nM) or vehicle and analyzed for CREB and TrkB phosphorylation. In some experiments hippocampal cultures were pretreated with DAAO (0.1 U/ml) and catalase (300 U/ml) for 1 h. For PARP analysis, hippocampal cultures were stimulated in Locke’s buffer for 30 min with NMDA (200 μM) alone or together with CX3CL1 (100 nM), washed, re-incubated in the previously stored conditioned medium and lysed after 16 h for western blot experiments. For each condition, equal amounts of proteins were loaded on SDS-polyacrylamide gel, band intensity was normalized for actin and densitometric analyses were performed with QuantityOne software (Biorad).

### RNA extraction and analysis for BDNF

Total RNA from primary hippocampal cultures (2.5 × 10^5^ cells) was extracted by Trizol reagent (Invitrogen). Reverse transcription reaction was performed in a thermocycler (MJ Mini Personal Thermal Cycler; Biorad) using IScript TM Reverse Transcription Supermix (Biorad; Milan, Italy) according to the manufacturer’s protocol. Real-time PCR (RT-PCR) was carried out in a I-Cycler IQ Multicolor RT-PCR Detection System (Biorad) using SsoFast EvaGreen Supermix (Biorad) according to the manufacturer’s instructions. The PCR protocol consisted of 40 cycles of denaturation at 95°C for 30 s and annealing/extension at 58°C for 30 s. For quantification analysis the comparative Threshold Cycle (Ct) method was used. The Ct values from bdnf gene were normalized to the Ct value of Gapdh in the same RNA samples. Relative quantification was performed using the 2^−ΔΔCt^ method (Schmittgen and Livak, 2008) and expressed as fold change in arbitrary values. Primer sequences targeted against mouse BDNF and mouse GAPDH were as follows: *bdnf* 5’-TGAGTCTCCAGGACAGCAAA-3’ (F); 5’-TGTCCGTGGACGTTTACTTCT-3’ (R); *gapdh* 5’-TCGTCCCGTAGACAAAATGG-3’ (F); 5’-TTGAGGTCAATGAAGGGGTC-3’ (R).

### Measurement of BDNF by ELISA

Hippocampal cultures were stimulated with CX3CL1 (100 nM) for 5, 30, 60 and 180 min and lysed with an ELISA lysis buffer (137 mM NaCl, 20 mM Tris-HCl, 1% NP40, 10% glycerol, 1 mM PMSF, 10 mg/ml aprotinin, 1 mg/ml leupeptin and 0.5 mM sodium vanadate). The cell debris were centrifuged and the supernatant was collected and analyzed for BDNF concentration (ng/ml) using the BDNF E_MAX_ ImmunoAssay System (Promega; Madison, Wi, USA). The ELISA was performed according to the manufacturer’s instructions.

### Statistical data analysis

Data are shown as the mean ± SEM; appropriate Student’s *t*-test or analysis of variance (ANOVA) were used as specified in figure legends. The significant differences between groups of data were specified in each figure legend. All statistical analysis was done using Sigma Plot 11.0 software.

## Results

### CX3CL1 neuroprotection against NMDA requires A_2A_R

**Figure 1:**
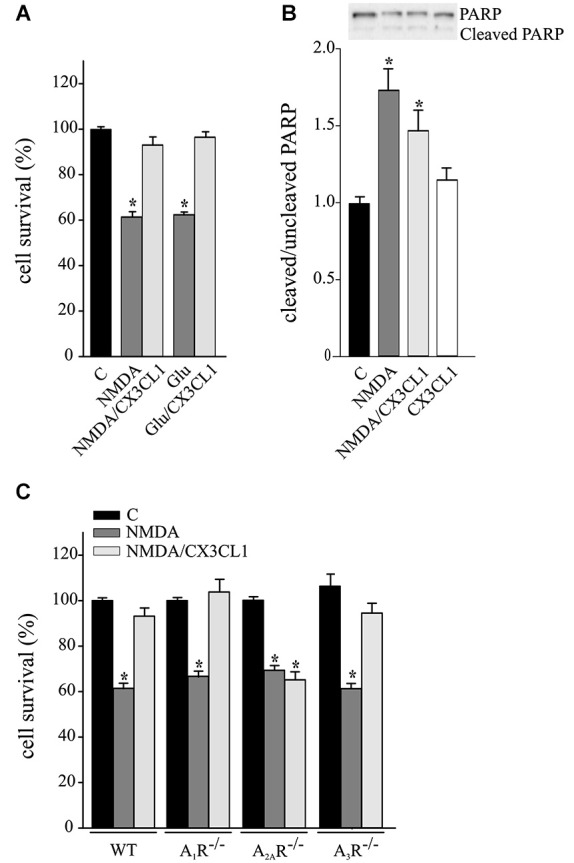
**Neuroprotective effect of CX3CL1 against NMDA toxic insult requires A_2A_R. (A)** Glu- and NMDA-excitotoxic experiments were performed in mouse hippocampal cultures in the presence or in the absence of CX3CL1 (*n* = 6–13). **(B)** Western blot analysis for PARP (cleaved and uncleaved PARP) in NMDA, NMDA/CX3CL1 and CX3CL1 treated hippocampal cells. Representative blots are shown on top. Data are expressed as % of cleaved/uncleaved PARP vs. untreated cells (C) (*n* = 3). **(C)** NMDA-excitotoxic experiments were performed in hippocampal cultures from WT, A_1_R^−/−^, A_2A_R^−/−^ and A_3_R^−/−^ mice treated or not with CX3CL1(*n* = 5–10). In **(A)** and **(C)** data are expressed as percentage of cell survival taking as 100% the number of viable cells in control condition (C). Results represent the mean ± SEM. Statistical analysis: one-way ANOVA followed by Dunn’s *post hoc* test *p ≤ 0.05 **(A)**; one-way ANOVA followed by Holm-Sidak *post hoc* test *p ≤ 0.05 **(B, C)**.

CX3CL1 is neuroprotective against Glu-induced excitotoxicity, with mechanisms fully dependent on A_1_R (Lauro et al., 2008, 2010) and in part dependent on A_3_R (Rosito et al., 2014). CX3CL1 also protects neurons against NMDA-induced death (Deiva et al., 2004), but no evidence of ARs involvement are reported so far. For this reason we performed experiments on the neuroprotective effects of CX3CL1 against NMDA- and Glu-induced excitotoxicity in primary hippocampal cultures. Data reported in Figure 1A confirms that CX3CL1 has similar protective effects both against NMDA (200 μM, 30 min) and Glu (100 μm, 30 min) (*n* = 6–13; *p* ≤ 0.05). CX3CL1 neuroprotective effect was also confirmed by MTT assay, being cell viability 57.6 ± 2.6% of control upon NMDA treatment (*p* ≤ 0.05) and rising to 91.6 ± 3% of control upon NMDA/CX3CL1 costimulation (*n* = 3; One way Anova followed by Holm-Sidak *post hoc* test). Similar results were obtained measuring cell death by LDH release: increasing to 139.4 ± 6.9% of control upon NMDA treatment (*p* ≤ 0.05) and being 104.4 ± 3.5% of control in NMDA/CX3CL1 treated cells (*n* = 3; One way Anova followed by Holm-Sidak *post hoc* test). To evaluate the contribution of apoptotic pathways, we analyzed the level of PARP cleavage and found that although a partial reduction between NMDA and NMDA/CX3CL1 treated cells was observed, it was not statistical significant (Figure 1B). All together these results are in line with the observation that both necrosis and apoptosis contribute to excitotoxic neuronal death (Bonfoco et al., 1995; Nicotera et al., 1999) and suggest that CX3CL1 plays effects on both pathways.

To evaluate the involvement of ARs in CX3CL1-mediated protection against NMDA, experiments were performed in primary hippocampal cultures from A_1_R^−/−^, A_2A_R^−/−^ and A_3_R^−/−^ mice. Results shown in Figure 1C demonstrate that the CX3CL1 protective effect was lost only in cell cultures lacking A_2A_R (*n* = 5–10; *p* ≤ 0.05).

### Extracellular D-serine mediates CX3CL1 neuroprotection against NMDA

**Figure 2:**
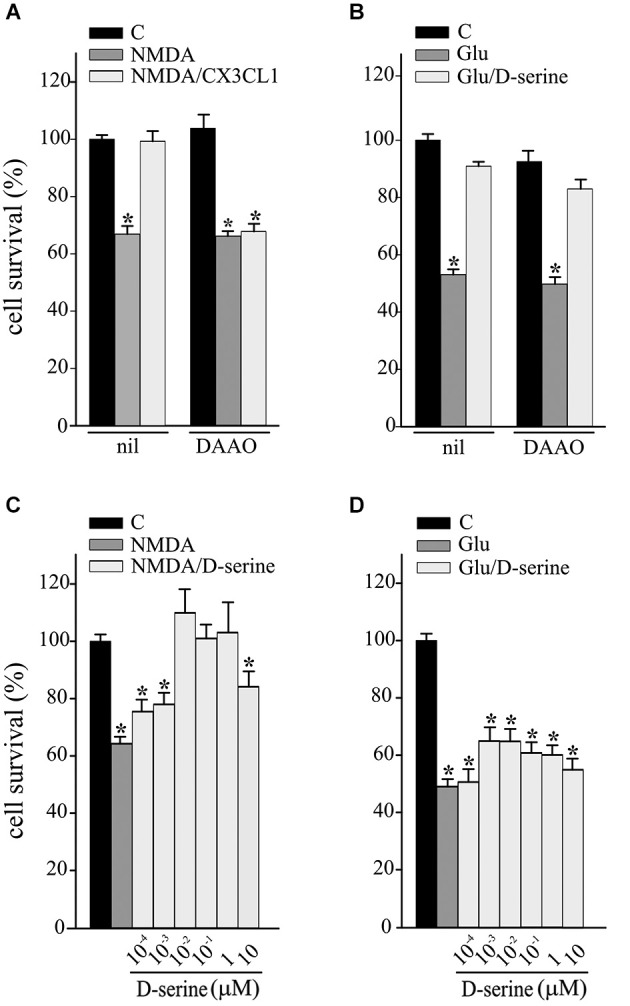
**D-serine contributes to CX3CL1 neuroprotection against NMDA toxic insult. (A)** NMDA- or **(B)** Glu-excitotoxic experiments were performed in mouse hippocampal cultures pretreated or not with DAAO/catalase (DAAO) and stimulated or not with CX3CL1 (*n* = 9 and *n* = 6 respectively). Data are expressed as percentage of cell survival in treated vs. untreated cultures (C) without DAAO. Results represent the mean ± SEM. Statistical analysis: one-way ANOVA followed by Holm-Sidak *post hoc* test *p ≤ 0.05 **(A)**; one-way ANOVA followed by Dunn’s *post hoc* test *p ≤ 0.05 **(B). (C)** NMDA- or **(D)** Glu-excitotoxic experiments were performed in mouse hippocampal cultures treated or not with D-serine, at the indicated concentration (*n* = 5–15 and *n* = 6–9 respectively). Data are expressed as percentage of cell survival in treated vs. C cultures without DAAO. Results represent the mean ± SEM. Statistical analysis: one-way ANOVA followed by Dunn’s *post hoc* test *p ≤ 0.05 **(C)**; one-way ANOVA followed by Holm-Sidak *post hoc* test *p ≤ 0.05 **(D)**.

We have recently demonstrated that CX3CL1 induces D-serine release by glial cells and that both D-serine and A_2A_R are necessary for CX3CL1-mediated potentiation of synaptic NMDAR function (Scianni et al., 2013). To investigate the possible involvement of D-serine in the neuroprotective effects of CX3CL1, excitotoxicity experiments were performed in the presence of DAAO (0.1 U/ml) and catalase (300 U/ml), to remove extracellular serine and the hydrogen peroxide by-products, respectively. Data reported in Figure 2 show that DAAO/catalase treatment (DAAO) efficiently inhibited the protective effects of CX3CL1 against NMDA-induced cell death (*n* = 9; *p* ≤ 0.05; Figure 2A), being ineffective on Glu-excitotoxicity (*n* = 6; *p* ≤ 0.05; Figure 2B). We also tested the ability of D-serine to promote neuroprotection against excitotoxicity: as reported in Figure 2C, upon NMDA challenge D-serine induced neuroprotection in a dose dependent way (*n* = 5–15; *p* ≤ 0.05) while it was ineffective in counteracting the effect of Glu challenge (*n* = 6–9; *p* ≤ 0.05 Figure 2D).

### CX3CL1 and D-serine induce CREB phosphorylation requiring the expression of A_2A_R

**Figure 3:**
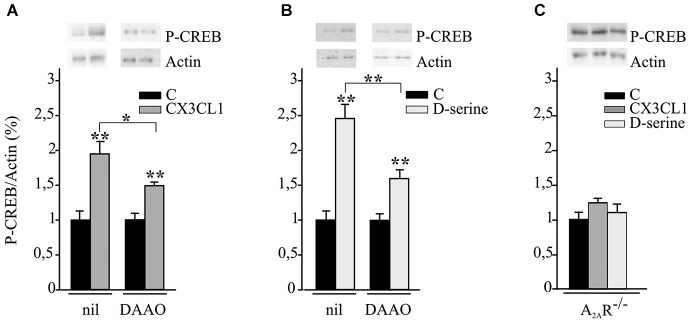
**CX3CL1 and D-serine induce CREB phosphorylation requiring the expression of A_2A_R. (A)** Mouse hippocampal cultures, pretreated or not with DAAO/catalase (DAAO), were analyzed by western blot for phospho-CREB (p-CREB) and actin upon CX3CL1 or **(B)** D-serine stimulation. Representative blots are shown on top. Data, expressed as % of p-CREB vs. C and normalized on actin, represent the mean ± SEM of 5 independent experiments. Statistical analysis: Student’s *t*-test **p ≤ 0.001 and one-way ANOVA followed by Holm-Sidak *post hoc* test *p ≤ 0.05 **(A)**; Student’s *t*-test **p ≤ 0.001 and one-way ANOVA followed by Dunn’s *post hoc* test **p ≤ 0.001 **(B). (C)** Hippocampal cultures from A_2A_R^−/−^ mice were analyzed by western blot for phospho-CREB and actin upon CX3CL1 or D-serine stimulation. Data, expressed as % of p-CREB vs. C and normalized on actin, represent the mean ± SEM of 5 independent experiments.

Hippocampal neuronal cultures were treated with CX3CL1 or D-serine for 15 min and analyzed for CREB phosphorylation. Data reported in Figures 3A,B demonstrate that both CX3CL1 and D-serine significantly increased CREB phosphorylation (*n* = 5; **p ≤ 0.001) and that DAAO/catalase treatment significantly reduced it (*n* = 5 *p ≤ 0.05 and **p ≤ 0.001).

Since it is known that NMDARs are able to modulate BDNF expression through CREB signaling (Hardingham et al., 2002; Jiang et al., 2005) we analyzed BDNF gene and protein expression upon CX3CL1 treatment. Data obtained indicate that both mRNA and protein BDNF levels were increased upon CX3CL1 treatment, with different kinetics. mRNA levels reach a peak at 15 min (2.7 ± 0.5 fold; *n* = 3; *p* ≤ 0.05) and are maintained up to 180 min (2.3 ± 0.4 fold; *n* = 3; *p* ≤ 0.05, One way Anova followed by Holm-Sidak *post hoc* test). BDNF protein is significantly increased after 60 min of CX3CL1 stimulation (time 0: 229.4 ± 1 pg/ml; 60 min: 754.5 ± 32 pg/ml; *n* = 3; *p* ≤ 0.05, One way Anova followed by Holm-Sidak *post hoc* test) and returns to basal values after 180 min (231.2 ± 9.6 pg/ml). Moreover we also observed that after 15 min of CX3CL1 stimulation there is an increase in TrkB phosphorylation (1.8 ± 0.3 fold; *n* = 3; *p* ≤ 0.05, One-way ANOVA followed by Dunn’s *post hoc* test).

**Figure 4:**
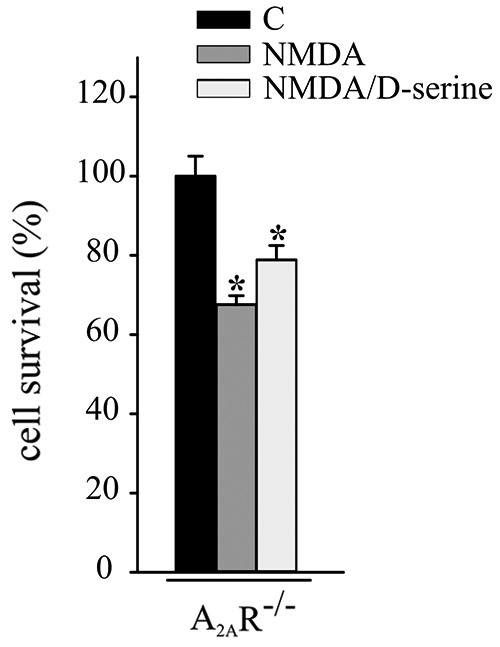
**A_2A_R mediates the neuroprotective effect of D-serine against NMDA-induced excitotoxicity**. NMDA-excitotoxic experiments were performed in hippocampal cultures from A_2A_R^−/−^ mice treated or not with CX3CL1 (*n* = 12) or D-serine (*n* = 10). Data are expressed as cell survival vs. C. Results represent the mean ± SEM. Statistical analysis: one-way ANOVA followed by Dunn’s *post hoc* test *p ≤ 0.05.

Experiments were performed to evaluate the role of A_2A_Rs in CX3CL1- and D-serine-mediated CREB phosphorylation. At this aim hippocampal neuronal cultures from A_2A_R^−/−^ mice were treated with CX3CL1 or D-serine for 15 min and analyzed for CREB phosphorylation. Interestingly, in the absence of A_2A_R, neither CX3CL1 nor D-serine were able to induce CREB phosphorylation (*n* = 7; Figure 3C). In order to understand if A_2A_R could play a role in mediating the neuroprotective effect of D-serine against NMDA-induced excitotoxicity, experiments were performed on A_2A_R^−/−^ hippocampal cultures in the presence of D-serine (100 nM). Results shown in Figure 4 demonstrated that in absence of the A_2A_R, D-serine has no neuroprotective effects (*n* = 10; *p* ≤ 0.05).

## Discussion

NMDARs are versatile receptors modulating physiological and pathological activities in the nervous system. They are involved in development, synapse maturation, synaptic transmission and plasticity processes, and their dysfunction has been implicated in several neurodegenerative diseases and mood disorders (Malenka and Bear, 2004; Hardingham and Bading, 2010; Zhou and Sheng, 2013; Ghasemi et al., 2014). NMDAR stimulation may induce neuronal death (Choi, 1987; Choi et al., 1988; Tymianski et al., 1993) or protection, also through preconditioning (Grabb and Choi, 1999; Navon et al., 2012). This apparently dicothomic behavior of NMDARs has been in part ascribed to their synaptic or extrasynaptic localization (Ikonomidou and Turski, 2002; Hardingham, 2006; Hetman and Kharebava, 2006), corresponding to different subunit composition (Hardingham et al., 2002; Chen et al., 2008). While synaptic NR2A/NMDARs are involved in protective mechanisms, extrasynaptic, NR2B/NMDARs preferentially lead to the activation of death pathways (Hardingham and Bading, 2010). D-serine is a preferential co-agonist of NR2A-containing NMDARs, and we wanted to verify the hypothesis that it could be involved in CX3CL1-induced neuroprotective effects. Although *in vitro* systems do not allow to distinguish between synaptic and extrasynaptic NMDAR, cultured hippocampal neurons do express both NR2A and NR2B subunits (Li et al., 2014) and can be used to investigate the effects of both kinds of NMDARs. In this paper we report data showing that: (i) NMDA-induced excitotoxicity is prevented by CX3CL1 with mechanisms involving A_2A_R; (ii) D-serine mediates the neuroprotective effect of CX3CL1 against NMDA- but not Glu-toxicity; (iii) CX3CL1 and D-serine phosphorylate CREB with a mechanism involving the presence of A_2A_R. Adenosine is a metabolite that acts through different receptor subtypes (Jacobson and Gao, 2006); among them A_1_R (Lauro et al., 2008, 2010) and A_3_R (Rosito et al., 2014) are involved in CX3CL1 neuroprotection against Glu-induced toxicity. Moreover we previously demonstrated that CX3CL1 triggers the release of adenosine from neuronal and microglial cells (Lauro et al., 2008, 2010). In this paper we demonstrate that upon NMDA-induced toxicity, the protective effects of CX3CL1 specifically requires A_2A_R expression and the presence of D-serine. These results are in line with the data showing that both CX3CL1-induced modulation of NMDA fEPSP and D-serine release from glial cell require A_2A_R (Scianni et al., 2013). Together these data suggest a mechanism where the protective effects of CX3CL1 are mediated by the activation and the expression of A_2A_R and consequentially the D-serine-dependent activation of NR2A/NMDARs. CX3CL1 modulates NMDAR effects, in an A_2A_R-dependent way, potentiating neurotransmission and increasing NR2A/NMDAR protective effects, likely shifting the balance of NMDAR activation toward NR2A-containing, D-serine-sensitive NMDARs. Nevertheless we cannot exclude that other pathways triggered by CX3CL1 act synergistically with A_2A_R: we showed indeed that CX3CL1 increases BDNF expression and TrkB phosphorylation, events possibly linked to CREB phosphorylation (Pizzorusso et al., 2000; Lee et al., 2005) thus explaining the partial block of CREB phosphorylation when D-serine is degraded by DAAO. We also speculate that at least part of the protective effects of CX3CL1 against NMDA could be due to A_2A_R-dependent intracellular pathways contrasting NMDA-mediated toxicity that do not depend on D-serine. Indeed, in addition to play a significant role in D-serine release, A_2A_R directly contributes to NR2A/NMDAR potentiation as shown by the observation that D-serine alone is not sufficient to induce neuroprotection or CREB phosphorylation in A_2A_R^−/−^ hippocampal cultures. These data may suggest that A_2A_R also acts downstream of D-serine, regulating the activity of NMDAR independently of D-serine. It should be considered that different populations of A_2A_R have different localization and functions, with those expressed by glial cells mostly responsible for the release of D-serine and those expressed post-synaptically modulating NMDARs activity. In line with this hypothesis, it has been reported that A_2A_Rs colocalize post-synaptically with metabotropic Glu receptors, playing a permissive role in mGluR5-mediated potentiation of NMDA in the hippocampus (Tebano et al., 2005). Alternatively A_2A_R could have a role in modulating NMDAR subunit phosphorylation, since NMDAR functions are known to be modulated by phosphorylation upon G-protein-coupled receptor activation (Köles et al., 2001). Our data suggest a functional interaction of NMDAR with A_2A_R, as shown in Huntington’s disease (Martire et al., 2010) and ischemia (Yang et al., 2013). The activation of A_2A_R induces the release of D-serine which acts on NR2A/NMDARs contributing to the enhancement of “CREB on” activation pathway (Hardingham and Bading, 2010). Part of the A_2A_R-mediated effect, however, is D-serine independent, and merits further investigation in this system. This is the first demonstration of a functional interaction between NMDAR and A_2A_R aimed at modulating protective effects: our data suggest the hypothesis that neuronal death induced by NMDAR activation could be counterbalanced by the selective activation (or potentiation) of synaptic, D-serine sensitive NR2A/ NMDARs, providing a possible therapeutic target for neurodegenerative pathologies. In conclusion, we provide evidence that CX3CL1 has a broad spectrum of protective activities against excitotoxicity induced by Glu-receptor activation, engaging different AR subtypes on neighboring cell types and efficiently contrasting neuronal cell death.

## Conflict of interest statement

The authors declare that the research was conducted in the absence of any commercial or financial relationships that could be construed as a potential conflict of interest.
